# Elastic Modulus of Osteoporotic Mouse Femur Based on Femoral Head Compression Test

**DOI:** 10.1155/2017/7201769

**Published:** 2017-12-10

**Authors:** Chang-soo Chon, Hui-suk Yun, Han Sung Kim, Cheolwoong Ko

**Affiliations:** ^1^Department of Biomedical Engineering, Yonsei University, 1 Yeonsedae-gil, Heungeop-myeon, Wonju-si, Gangwon-do 26493, Republic of Korea; ^2^Power & Ceramic Division, Korea Institute of Materials Science (KIMS), 797 Changwon-daero, Seongsan-gu, Changwon-si, Gyeongsangnam-do 51508, Republic of Korea; ^3^Biomedical System & Technology Group, Korea Institute of Industrial Technology (KITECH), 89 Yangdaegiro-gil, Ipjang-myeon, Seobuk-gu, Cheonan-si, Chungcheongnam-do 31056, Republic of Korea

## Abstract

A biomechanical test is a good evaluation method that describes the structural, functional, and pathological differences in the bones, such as osteoporosis and fracture. The tensile test, compression test, and bending test are generally performed to evaluate the elastic modulus of the bone using mice. In particular, the femoral head compression test is mainly used for verifying the osteoporosis change of the femoral neck. This study conducted bone mineral density analysis using in vivo microcomputed tomography (micro-CT) to observe changes in osteoporosis over time. It proposed a method of identifying the elastic modulus of the femur in the normal group (CON group) and the osteoporotic group (OVX group) through finite element analysis based on the femoral head compression test and also conducted a comparative analysis of the results. Through the femoral head compression test, it was verified that the CON group's ultimate and yield loads were significantly higher than those of the OVX group. It was considered that this result was caused by the fact that the bone mineral density change by osteoporosis occurred in the proximal end more often than in the femur diaphysis. However, the elastic modulus derived from the finite element analysis showed no significant difference between the two groups.

## 1. Introduction

As the aging of the population has increased due to the development of medical technology, the frequency of fracture patients is gradually increasing [1]. In particular, femur intertrochanteric and femoral neck fractures are common in elderly patients. These fractures are caused by falls, traffic accidents, industrial accidents, and so forth [2]. The fractures are closely connected with death [3], and the mortality rate within one year following the fracture is reported as 14–36% [4]. As there are many cases of femur fracture patients with bone fragmentation and osteoporosis, it is difficult to obtain stable internal fixation during the treatment [5].

The treatment of most femur fracture patients requires surgery with internal fracture fixation using intramedullary nails and bone plates. Therefore, understanding the biomechanical characteristics, such as load distribution, micromovement, and bone strength change affecting the fixation force of the implants used for the internal fixation, in particular, including the elastic modulus of the bone, provides important information regarding recovery and rehabilitation [6]. The elastic modulus of the bone has been mentioned as one of the most important elements for bone evaluation. The material property evaluation of the bone through the mechanical strength test is known as the best method [7]. The biomechanical test is an effective method for explaining the structural, functional, and pathological differences in osteoporosis and fractures. Various biomechanical studies have been conducted, for example, observing changes by causing osteoporosis in mice or carrying out strength tests by extracting the bone [8, 9].

A three-point bending test and four-point bending test are mainly used as evaluation methods for the elastic modulus of the bone in mice [7]. According to previous studies, in the case of a long bone such as the femur and tibia, osteoporosis often occurs from the proximal and distal positions. Moreover, it is known that the change in the diaphysis is imperceptible [10]. In addition, as the diaphysis in the femur mainly consists of the cortical bone, it is difficult to accurately understand the structural elastic modulus of the whole bone or the fracture accompanying osteoporosis. Therefore, elastic modulus analysis through a femoral head compression test is required to understand the femoral neck fracture and verify the effect of osteoporosis on the bone.

This study restructured the shape of the femur and analyzed changes in bone mineral density (BMD) by time based on the micro-CT images of the femur after breeding 12-week-old mice for 20 weeks in the OVX group and CON group. In addition, the femoral head compression test was carried out by extracting the femur, and the elastic modulus of the femur was identified through femoral head compression finite element analysis. Then, the comparison and analysis of the femur elastic modulus between the OVX group and the CON group were carried out.

## 2. Methods

### 2.1. Animal Testing

**Figure 1:**
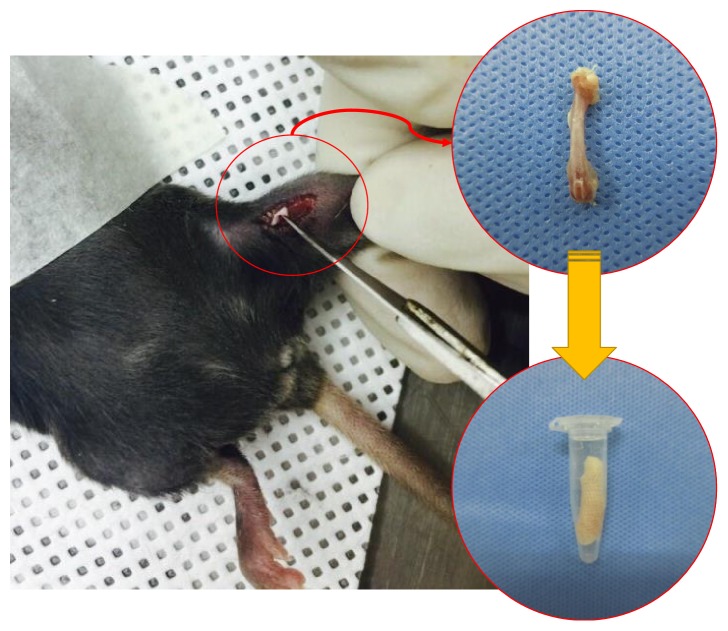
The right femurs were extracted for the femoral head compression test.

The experimental animals used in this study were 20 healthy female C57BL/6 mice at the age of 12 weeks (Samtako, Republic of Korea) (21.5 ± 1.3 g). They were divided into two groups of 10 each: the osteoporotic group (OVX group) in which osteoporosis was caused through ovariectomy and 10 in the normal group (CON group). They were bred for 20 weeks. The animal breeding room maintained a constant temperature (23 ± 3°) and humidity (50 ± 5%). The animals were bred, separated into a cage, and allowed free intake of water and food. At an interval of 4 weeks up to 20 weeks after the ovariectomy, the right femur was scanned using a micro-CT (Skyscan 1176, Bruker, Belgium) (Figure 1). The bone mineral density was measured through three-dimensional (3D) reconstruction of the femur based on the micro-CT image. The animal test in this study complied with the Guidelines and Rules on the Protection and Use of Animals of Yonsei University (YWC-150126-1).

### 2.2. In Vivo Micro-CT

In the micro-CT scan, the voxel size was set in 18 *μ*m; the filter, aluminum 0.5 mm; the exposed time, 210 ms; the voltage, 55 kV; the current, 455 *μ*A; and the rotation angle, 0.5°. The experimental animals were anesthetized with gas for the scan. The anesthetic, isoflurane (Ifran Liquid, JW Pharmaceutical, South Korea; isoflurane 5 vol% O2–1.3 L/min used per mouse for initial anesthesia and isoflurane 1.4 vol% O2–1.3 L/min used per mouse for anesthesia maintenance) was used. The right femur was extracted from the OVX group and CON group for the femoral head compression test after the scan (Figure 1).

### 2.3. Femoral Head Compression Test

**Figure 2:**
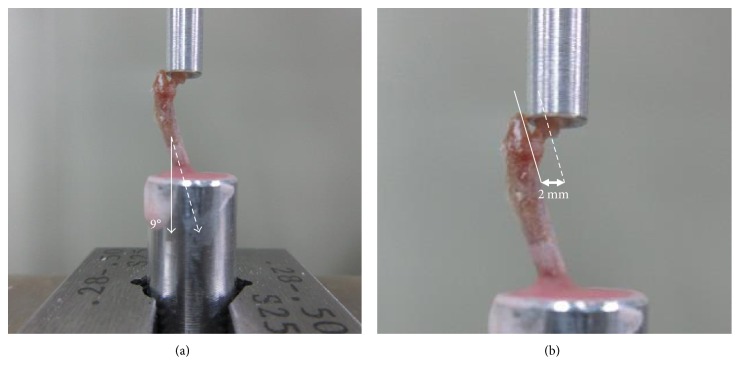
Setup of the femoral head compression test. (a) The femur was potted in a cylindrical lower jig at a 9° angle. (b) The gage length is the distance from the center point of the femoral head to the medial surface of the diaphysis.

**Figure 3:**
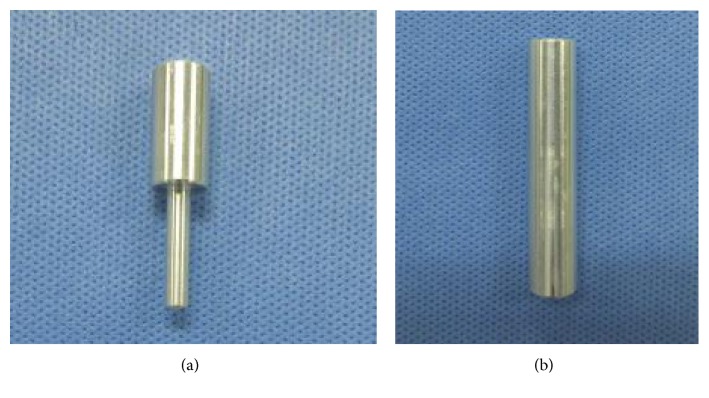
Femoral head compression test jig. (a) Upper jig. (b) Lower jig.

With 10 femurs of the OVX group and 10 femurs of the CON group extracted from the 32-week-old mice after breeding for 20 weeks, a femoral head compression test was conducted, using an Instron E3000 (Norwood, MA, USA). Femur specimens were kept in a freezer at −20°C for 2 weeks after harvesting and stored in a saline solution before the test. Referring to the literature for the anatomical axis of the femur [11], a femoral head compression test was set up using upper and lower jigs, vertical in the sagittal plane and extroverted at 9° in the coronal plane, with the femoral specimens. The lower part of the femur was completely fixed in the lower jig using methyl methacrylate (VertexTM Self-Curing, Vertex-Dental B.V., Zeist, Netherlands) (Figure 2). The upper jig that contacted the femoral head was designed in the hemispheric shape with a radius of 2 mm, considering the shape of the femoral head. The upper jig was produced to avoid any interference with the femur diaphysis during the compression test (Figure 3).

**Figure 4:**
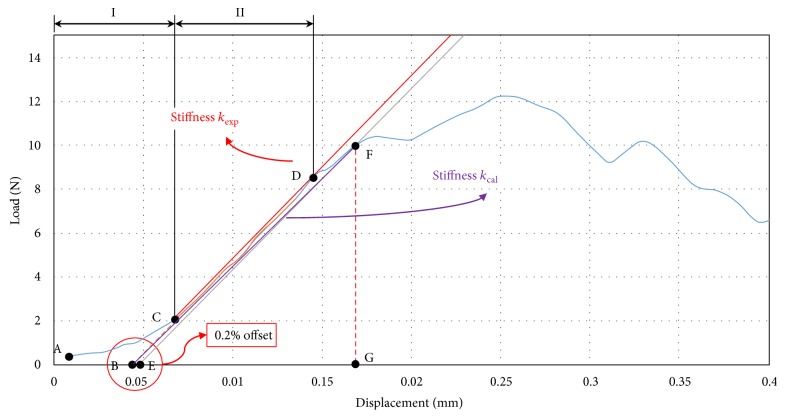
Load-displacement curve for the femoral head compression test of the CON group. Two characteristic curve regions are marked for load-displacement curve: (I) increasing contact region of the upper jig with the femoral head and (II) nearly linear region.

A compression load was applied at a rate of 10 mm/min. The compression test was performed until the femur specimens fractured or the load generated decreased by more than 20% from the ultimate load (Figure 2). The load-displacement curve obtained in the compression test was recorded at an interval of 4 Hz, using Instron Dynacell 1kN load cell. From the test results, ultimate load (N), 0.2% yield load (N), and stiffness *k*_exp_ (N/mm) were calculated (Figure 4).

The explanation regarding the load-displacement curve obtained through Figure 4 compression test is as follows: first, in accordance with the American Society of Testing and Materials (ASTM D790-03), section I (A–C) of the load-displacement is assumed as the toe section where the contact between the femoral head and the upper jig increased, and load-displacement curve was compensated. In addition, from point C, where the toe section ended, section II (C-D) where the load-displacement curve appeared in a linear condition was set, and stiffness *k*_exp_ (N/mm) was identified. In addition, the yield load in the elastic region was identified using a 0.2% offset method [12]. The offset was set to 0.2% (0.004 mm) of the lever arm (distance from the femoral head central point to the femur diaphysis in the compression test: 2 mm) in accordance with the American Society of Testing and Materials (ASTM F384).

(1) $$\begin{array}{c} 0.2\%\;offset=lever\;arm\;\left(2\;mm\right)\times 0.002\;\left[mm\right]. \end{array}$$

### 2.4. Femoral Head Compression FE Analysis

**Figure 5:**
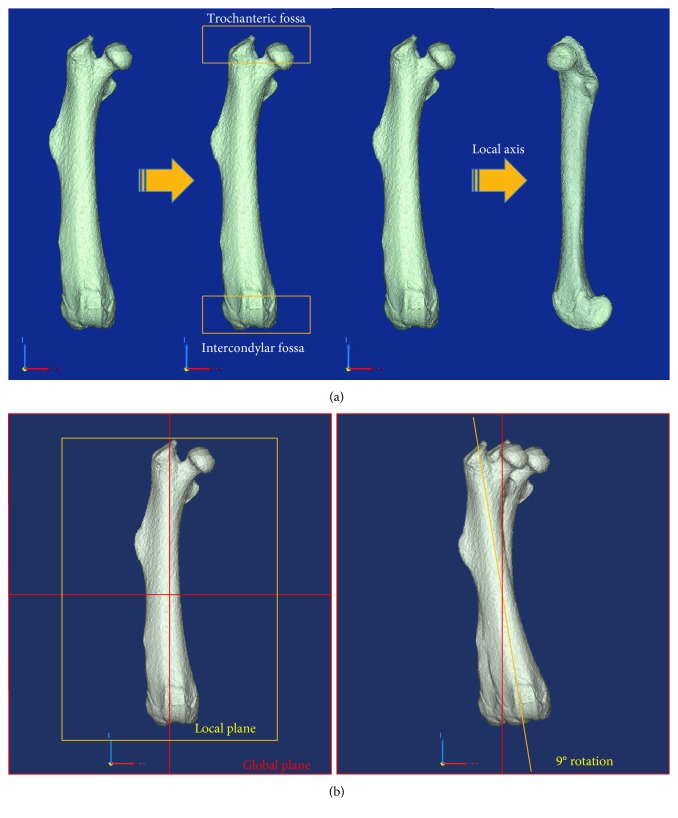
The schematic illustrations of the rearrangement of a femur for finite element analysis. (a) The local *z*-axis was aligned with the global *Z*-axis. (b) The local x-z plane was aligned with the global X-Z plane, and the femur was rotated 9° in the global X-Z plane.

For the finite element analysis, a 3D finite element model was constructed based on the 2D micro-CT cross-sectional images of the OVX group and CON group on week 0 and week 20 from the beginning of the test (age of 12 weeks) using 3D modeling software Mimics 18.0 (Materialise, Leuven, Belgium) (Figure 5). In the 3D modeling of the femur, a range of 226–3071 was applied as the Hounsfield unit (HU) value, and the bone mineral density (*ρ*) of the femur was calculated by the phantom formula used in the micro-CT scan [13].

(2) $$\begin{array}{c} \rho =0.00023\times HU-0.0266\;\left[g/cm^{3}\right]. \end{array}$$

**Figure 6:**
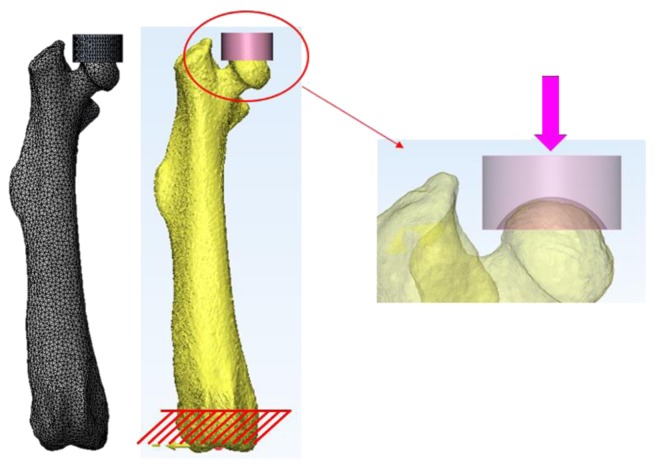
Load and boundary conditions for finite element analysis.

3D femur models, constructed for head compression analysis of the femur in the same condition as that of the femoral head compression test, were relocated based on the same coordinate system (Figure 5). In addition, in order to apply the same test conditions as that of the compression test, the femur models were placed vertically in the sagittal plane and extroverted at 9° in the coronal plane [11]. The upper jig model was constructed in the same way as the upper jig contacting the femoral head in the compression test (Figure 6).

For elastic modulus identification through a finite element analysis, the elastic region up to the yield load of the compression test was applied. The region (B–F) connecting point B and point F was set to the elastic region and defined as stiffness *k*_cal_ (Figure 4).

The elastic modulus of the femur applied to the finite element analysis was defined as nonlinear interaction equation (3) [14–16]. A finite element analysis was conducted based on the calculated elastic modulus, applying the bone mineral density of each femur calculated from the micro-CT images and changing constants *A* and *B*. From the compression analysis result of each femur, the case in which the reaction force and displacement in the upper jig corresponded to the yield load (*F*) and yield displacement (*G*) was selected, and the elastic modulus was identified.

(3) $$\begin{array}{c} E=A\times \rho^{B}\;\left[MPa\right]. \end{array}$$

*E* is the elastic modulus, *ρ* is the bone mineral density, and *A* and *B* are constants.

A finite element analysis was conducted in a contact nonlinear analysis condition, and the friction coefficient between the upper jig model and the femur model was assumed to be 0.3, and Poisson's ratio of 0.3 [17] was applied. In addition, the lower section of the femur was completely fixed up to 2 mm from the bottom in the axial loading direction, the same as the fixing condition in the compression test. Tetrahedron was applied as the element type of the femur model and defined as a linear elastic body. Finite element models were meshed using an element size of 0.2~0.25 mm (approximately 68,000~71,000 elements). The upper jig model was set to a rigid body, and commercial software ANSYS 16.0 (ANSYS Inc., USA) was used as an analysis solver.

### 2.5. Statistical Analysis

A statistical analysis (*t*-test) was carried out using SPSS 23 (SPSS Inc., USA) to verify the difference in bone mineral density, ultimate load, yield load, yield displacement, stiffness, and elastic modulus, respectively (*p* < 0.05).

## 3. Result

### 3.1. BMD Based on Micro-CT Images

**Figure 7:**
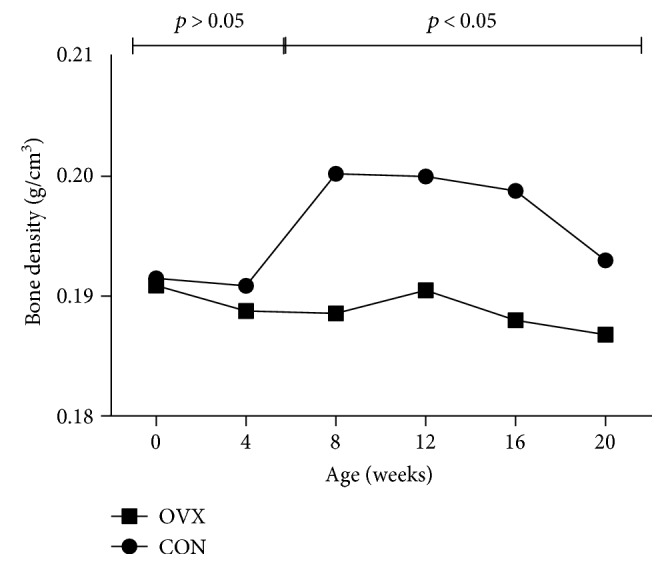
Bone mineral density by period from week 0 to week 20.

The bone mineral density measurement through the micro-CT scan of the OVX group and CON group during the 20-week breeding period of the 12-week-old mice revealed that the mean bone mineral density was similar between both groups between week 0 and week 4. There was no significant difference between the two groups. However, from week 8, the difference in the mean bone mineral density between the CON group and the OVX group rapidly increased. On week 8, there was a significant difference of 5.6%; week 12, 4.8%; week 16, 5.5%; and week 20, 3.2% (Figure 7) (Table 1). From these findings, it was considered that the induction of osteoporosis was found in the animal tests of this study.

### 3.2. Mechanical Bone Properties

**Figure 8:**
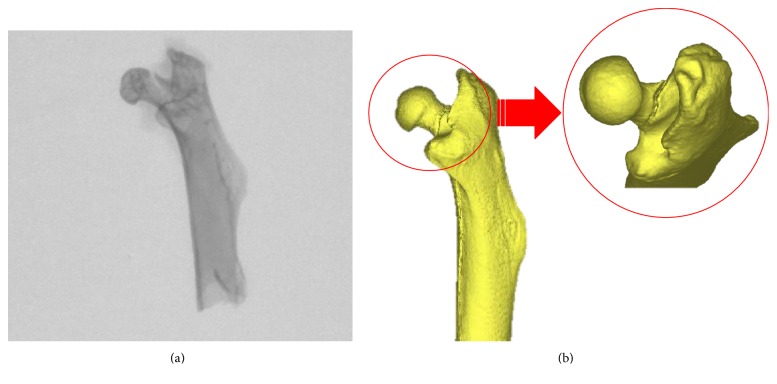
Example of femur neck fracture. (a) Micro-CT image. (b) 3D reconstruction image for femoral head.

**Figure 9:**
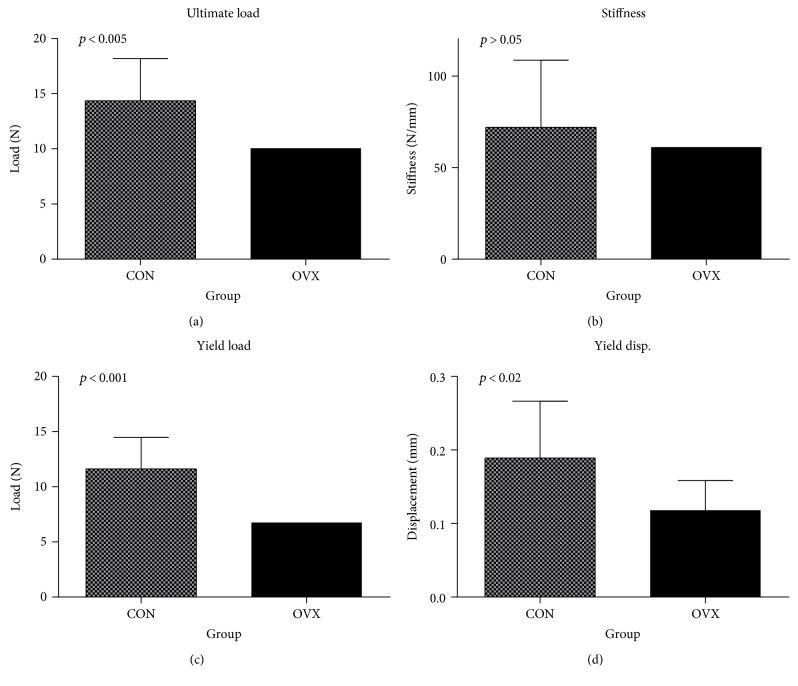
Results of mechanical bone properties. (a) Ultimate load, (b) experimental stiffness, (c) yield load, and (d) yield displacement for CON and OVX groups.

The femoral neck showed damage from both the CON group and the OVX group (Figure 8), and the result of a femoral head compression test with 32-week-old mice (week 20) is shown in Figure 9. The ultimate load and stiffness *k*_exp_ of the OVX group and CON group were calculated (Figure 9(a)). The mean ultimate load of the CON group was 14.36 N, and the OVX group decreased approximately 30% to 10.06 N. This showed a significant difference (*p* < 0.005).

The mean stiffness *k*_exp_ of the CON group was 72.04 N/mm. The OVX group showed a mean of 61.27 N/mm, which was lower by approximately 15% compared to that of the CON group (Figure 9(b)). However, there was no significant difference in the stiffness *k*_exp_ between the CON group and the OVX group (*p* > 0.05).

For the yield load (Figure 9(c)), the CON group showed the mean 11.62 N and the OVX group showed the mean 6.76 N. Thus, the OVX group decreased approximately 42% compared to the CON group, showing a significant difference (*p* < 0.001). In addition, the yield displacement of the OVX group (mean 0.12 mm) decreased approximately 38% compared to the CON group (mean 0.19 mm), showing a significant difference (*p* < 0.02) (Figure 9(d)).

### 3.3. Result of Femoral Head FE Analysis

In the finite element analysis based on the femoral head compression test (Figure 6), the elastic modulus of the femur of the OVX group and CON group was identified by a comparison of the yield load and yield displacement between the test result and the calculated result through (3), which defines the elastic modulus of the femur. The mean elastic modulus of the OVX group was 1600 MPa, and that of the CON group was 1683 MPa (Table 2). There were no significant differences in the mean elastic modulus between the two groups (*p* > 0.05).

The mean values of constants *A* and *B* applied for elastic modulus identification was 0.042 and 2.00, respectively, in the CON group, and 0.047 and 2.00, respectively, in the OVX group. The proposed equations of the mean bone mineral density and elastic modulus by each group are as follows:

(4) $$\begin{array}{c} E_{Con\_cal}=0.042\times \rho_{mean}^{2.00},\\ E_{OVX\_cal}=0.047\times \rho_{mean}^{2.00}. \end{array}$$

*E*_Con_cal_ is the elastic modulus calculated in the CON group, *E*_OVX_cal_ is the elastic modulus calculated in the OVX group, and *ρ*_mean_ is the mean bone mineral density.

Regarding the results of the calculation of the elastic modulus, when substituting the mean bone mineral density of the CON group (192 kg/m^3^) and OVX group (187 kg/m^3^) in the proposed equation (4), the elastic modulus calculated in the CON group was 1539 MPa, and the elastic modulus calculated in the OVX group was 1633 MPa. There was an approximately 3.8% error with the mean elastic modulus (1600 MPa) of the CON group obtained from the compression analysis. With the mean elastic modulus (1683 MPa) of the OVX group, there was a 3.0% error (Table 3).

## 4. Discussion

This study divided C57BL/6 mice into the OVX group and CON group and proposed an elastic modulus identification of the femur through micro-CT, a mechanical strength test, and finite element analysis. This study conducted a femoral head compression test in order to investigate the property change according to the bone mineral density difference. Through a finite element analysis, this study found no significant changes in the elastic modulus consistent with osteoporosis. In addition, this study proposed a bone mineral density-elastic modulus equation of the mouse femur and showed an error of less than 3.8% from the elastic modulus, calculated by applying the mean bone mineral density and elastic modulus identified through tests and finite element analysis.

### 4.1. Evaluation through Femoral Head Compression Test

According to Turner and Burr [7], because mice have a smaller bone size compared to the required specimen size, it is difficult to conduct a tensile and compression test. Thus, a bending test is used to evaluate the property of the long bone. The test measured the cross-section area of the diaphysis using 2D imaging equipment like micro-CT, drew the inertia moment, and calculated the elastic modulus by applying this to the proposed equation [18]. However, it is difficult to conduct a three-point bending test, four-point bending test, or torsion test of the long bone in order to evaluate the proximal or distal ends where most osteoporosis changes occur. According to Stürmer et al. [19], fractures due to the decrease in bone mineral density mostly occur in the metaphysis of the long bone. A bone mineral density measurement to evaluate the degree of osteoporosis is performed in the femoral neck or lumbar. Thus, based on an elastic modulus obtained through a bending test of the diaphysis with mainly cortical bone, there is a limitation in checking for changes in osteoporosis in the femur according to the compression load and changes in the cancellous bone. The distal femur mostly consists of cancellous bone. It has been reported that property changes in the bone appear noticeable consistent with osteoporosis [20]. In particular, most osteoporosis fractures are in the femoral neck and femur intertrochanteric [21–23]. Thus, in the human body, a femoral head compression test is used to investigate property changes according to the differences in bone mineral density [24, 25].

This study performed a head compression test of the right femur extracted from 32-week-old mice and found significant differences in the ultimate load and yield load between the OVX group and the CON group. It was considered that this was the impact of changes in bone mineral density consistent with osteoporosis as Ekeland et al. [26] and Keller et al. [27] reported. However, there was no significant difference in stiffness. This was because, as reported by Kamal et al. [11], the complex microstructure of the femoral neck was considered to limit the sensitivity of femoral neck test for stiffness.

### 4.2. Identification of Elastic Modulus through a Finite Element Analysis

This study performed a finite element analysis based on test results to identify an elastic modulus. However, in order to analyze the behavior of fracture injuries, it is necessary to include the plastic area as well as the elastic region. It is also necessary to conduct an analysis considering the anisotropic and inhomogeneous characteristics of the bone. In addition, for inhomogeneous bone mineral density, it is desirable to apply a different value to each region. However, this study conducted an analysis, applying the mean value of the entire femur for the bone mineral density of the finite element model to calculate an equation of the single bone mineral density. In the future, for a more accurate bone strength analysis, it will be necessary to conduct an analysis applying a different bone mineral density by region.

In previous studies of mice, mechanical strength tests were conducted in order to analyze the growth of mice or osteoporosis change over time. However, a bending test and torsion test calculated an elastic modulus in the cortical bone and used this to predict fractures and observed changes in osteoporosis. Thus, in bone material property evaluation for the prediction of fractures and observation of changes in osteoporosis, the type of load, distribution of bone mineral density, and load of the bone, physiologically and functionally applied, should be considered.

This study identified a more accurate elastic modulus of the CON group and OVX group of C57BL/6 female mice through a finite element analysis based on a femoral head compression test. It is considered that, in the future, through the method proposed in this study, it will be possible to investigate the changes in the elastic modulus according to changes in bone mineral density of the cancellous bone by period and predict fracture risks of both normal and osteoporosis bones of the femoral neck utilizing the derived findings.

## Figures and Tables

**Table 1:** Comparisons of femur mean bone densities in animal model.

BMD	CON group (mean)	OVX group (mean)	*p* value
0 week	0.192	0.191	0.86
4 weeks	0.193	0.190	0.74
8 weeks	0.200	0.189	0.01
12 weeks	0.199	0.191	0.01
16 weeks	0.196	0.188	0.001
20 weeks	0.192	0.187	0.05

Unit: g/cm^3^. *p* < 0.05.

**Table 2:** Comparison of Young's modulus in the CON group and OVX group.

	Mean density (kg/m^3^)	Mean *A*	Mean *B*	Mean Young's modulus (MPa)
CON group	192	0.042	2.00	1600
OVX group	187	0.047	2.00	1683

**Table 3:** Comparison of Young's modulus.

	Mean Young's modulus (MPa)	Calculated Young's modulus (MPa)	Error rate
CON group	1600	1539	3.8%
OVX group	1683	1633	3.0%

## Abbreviations

BMD: Bone mineral density
CON: Control group
Osteoporotic: Ovariectomy group
Micro-CT: Microcomputed tomography
*E*: Elastic modulus or Young's modulus
FE: Finite element
HU: Hounsfield unit
*k*_exp_: Experimental stiffness
*k*_cal_: Calculated stiffness
*ρ*: Bone mineral density
*ρ*_mean_: Mean bone mineral density
*E*_Con_cal_: Calculated elastic modulus of the control group
*E*_OVX_cal_: Calculated elastic modulus of the osteoporotic group.
